# Design of a Sensor System for On-Line Monitoring of Contact Pressure in Chalcographic Printing

**DOI:** 10.3390/s17092029

**Published:** 2017-09-05

**Authors:** José Antonio Jiménez, Francisco Javier Meca, Enrique Santiso, Pedro Martín

**Affiliations:** Department of Electronics, University of Alcalá, Alcalá de Henares, Madrid 28805, Spain; francisco.meca@uah.es (F.J.M.); enrique.santiso@uah.es (E.S.); pedro.martin@uah.es (P.M.)

**Keywords:** chalcography printer, roller alignment, contact pressure, gauge measurement, nip pressure, load cell, strain measurement

## Abstract

Chalcographic printer is the name given to a specific type of press which is used to transfer the printing of a metal-based engraved plate onto paper. The printing system consists of two rollers for pressing and carrying a metal plate onto which an engraved inked plate is placed. When the driving mechanism is operated, the pressure exerted by the rollers, also called contact pressure, allows the engraved image to be transferred into paper, thereby obtaining the final image. With the aim of ensuring the quality of the result, in terms of good and even transfer of ink, the contact pressure must be uniform. Nowadays, the strategies utilized to measure the pressure are implemented off-line, i.e., when the press machines are shut down for maintenance, which poses limitations. This paper proposes a novel sensor system aimed at monitoring the pressure exerted by the rollers on the engraved plate while chalcographic printer is operating, i.e., on-line. The purpose is two-fold: firstly, real-time monitoring reduces the number of breakdown repairs required, reduces machine downtime and reduces the number of low-quality engravings, which increases productivity and revenues; and secondly, the on-line monitoring and register of the process parameters allows the printing process to be reproducible even with changes in the environmental conditions or other factors such as the wear of the parts that constitute the mechanical system and a change in the dimensions of the printing materials. The proposed system consists of a strain gauge-based load cell and conditioning electronics to sense and treat the signals.

## 1. Introduction

Chalcography is the art of relief printing using rollers and metal plates among other components. The traditional chalcographic printers pose the problem that the contact pressure or mechanical stress in the roller-plate contact area, known as nip pressure, can be non-uniform along the roller generatrix. 

Contact pressure is applied by lowering the upper pressure drum onto the press bed using pressure adjusting screws [1]. Currently, there are no printing systems that include on-line monitoring of the contact pressure. It is always necessary to shut down the machines in order to make the appropriate adjustments to guarantee a high quality of printing. In this paper, the design of a sensor system aimed at monitoring the contact pressure in chalcographic printers for printing proofs is described. The system implemented allows the performance of various ink and paper types to be modeled and evaluated so that they can be used in production processes requiring gravure on an industrial scale, such as the printing of paper money and others.

The proper roller alignment ensures a uniform contact pressure, which is vital not only to obtain a high quality product but also to guarantee an efficient process, increasing yield and reducing waste in terms of ink and paper. An incorrect alignment can result in high costs due to the damage caused to both the chalcographic printers and the final product. The chalcographic printers include mechanical systems for micrometer alignment. However, they lack systems that enable the real pressure to be determined, and as a result, such pressure cannot be effectively controlled. 

For this reason, short-term reproducible pressure conditions are only possible and, therefore, when parts become worn or there is variation in the dimensions or properties of the printing materials, mainly ink and paper, the impression changes. By including the proposed monitoring sensor system, the adjustment does not depend on the above-mentioned factors, which allows the alignment condition of the chalcographic printer to be determined. As a result, the reproducibility of the impressions improves, i.e., there are hardly any deviations from one another, due to the fact that the long-term conditions (temperature and contact pressure) can be easily replicated. 

Therefore, in order to ensure a high-quality and efficient printing process, the measurement and control of the contact pressure generated by the rollers against the engraved plate is critical. Unfortunately, obtaining accurate measurements of the contact pressure is a challenging task, especially when the chalcographic printer is operating.

As stated above, the chalcographic printers currently available on the market include systems for precision adjustment [1]. However, there are no printers which have built-in electronics in order to online monitor the pressure exerted by the rollers on the engraved plate. Currently to this aim, external sensors are used instead.

Nowadays, there are two ways of measuring the contact pressure, namely: static and dynamic. The former is straightforward and it is carried out when the rollers are stationary. A static sensor is placed between the rollers or between the roller and the engraved plate and then some pressure is applied. If necessary, the pressure can be adjusted until the desired value is obtained and then it is measured in both sides and in the center of the roller. There are two systems based on static sensors: the traditional sensor films or nipimpression paper and the feeler gauges.

- The sensor films or nip impression paper [2,3,4] are based on a film that is placed between the roller and the engraved plate to characterize the contact pressure statically. Then, some pressure is applied with the rollers at rest. This strategy is not expensive although it can turn out to be costly depending on the size of the contact area and the number of measurements required until a proper alignment is achieved.
- The feeler gauges, as a mechanical tool, are usually used for thickness measurement purposes although they can also be used to measure the uniformity of the roller-plate contact area. Depending on the size of the contact area, this approach can be time-consuming. Besides, the information is incomplete: the feeler gauges can only identify potential defects between the roller and the plate and they cannot provide information about roller-plate misalignments.

Both the nip impression paper and the feeler gauges are not entirely accurate as they introduce a systematic error on account of the additional thickness of the sensor itself. 

There are also commercial digital meters which allow static measurements of the contact pressure. The strategy employed is similar to that of the Impression Paper, but they can show the corresponding measurement in display-based devices [5]. 

The dynamic measurement, on the other hand, is made with the rollers slowly rotating. Unlike the static measurement, the dynamic approach is more realistic since the shape and characteristics of the rollers change when they are subjected to pressure. Hence, this is the preferred method to employ when it comes to precisely controlling the contact pressure. In [6,7], two similar approaches to dynamic contact pressure assessment are presented, named sigma-nip and nip pressure alignment tool (NPAT), which are based on an array of pressure sensors. 

Both the static and dynamic techniques, explained previously, reveal an important limitation: they are procedures that are carried out when the printer is at rest, under programmed maintenance and hence they halt production. Besides, these systems do not take into account the thickness of the engraved plate and the paper, and some of them cannot work within the operating temperatures of the chalcographic printers. 

In this paper, a novel sensor system aimed at obtaining the pressure profile between two rollers or between one roller and the engraved plate is presented. The main contribution of this paper is that the pressure profile is generated while the printer is working, which guarantees a correct roller-plate alignment. This, in turn, results in a significant improvement in terms of printing quality, cost savings and long-term reproducibility of the engravings. 

The designed sensor is a custom load cell based on 16 strain gauges arranged in a 4 × 4 matrix configuration. It must be noted that locating strain gauges on the two outermost edges of the engraved plate would be enough to be able to detect the roller misalignment. However, in this work, the four edges have been used with the aim of obtaining additional information. This information can be used to detect potential deformations and the wear of the rollers and the engraved plate, which can produce wrong profiles in the contact pressure. 

The transducers based on strain gauges are inexpensive and provide high precision, long-term stability and sufficient response time to allow high-speed measurements [8]. Therefore, the strain gauge-based transducers are the best option for both high-scale industrial processes and more simple tasks, especially when it comes to high precision measurements. These features truly justify the widespread use of this kind of sensor in fields ranging from the healthcare sector [9,10,11] to the industrial sector [12,13,14,15] and civil engineering [16], among others.

In the last few years, several works have reported the use of pressure sensors based upon fiber optic sensors with promising results. The main drawback of fiber optic sensors, however, is the high cost associated with them. Hence, their use is restricted to special applications usually related to the construction and infrastructure sector [17,18,19,20].

Taking into account that the ink characteristics vary with the temperature, the proposed sensor includes a control system aimed at maintaining a constant temperature in the plate surface, usually 80 °C, which is the normal process temperature in the industrial chalcographic printers. However, the current test regime for chalcographic printers does not allow tests of chalcographic printing at a temperature of 80 °C to be carried out. The proposed system presented in this paper includes a heating module for the engraved plate with a corresponding temperature control unit. One of the most relevant features of the heating module is the low energy cost required to maintain the normal operating temperature of the engraved plate. This is possible on account of the module size, which allows the module to be located underneath the engraved plate, thereby minimizing heat loss. When implemented, the traditional technique is based on cartridge heaters of considerable dimensions, which cannot be placed close to the engraved plate. This fact causes substantial heat loss in comparison with the heating strategy developed in this paper, which is described in the following section.

To conclude, the proposed system automatically generates a detailed report with the data captured in each impression, namely: the pressure profile of the roller against the engraved plate, the temperature of the engraved plate and hence that of the ink used in the printing process, environmental temperature and humidity and finally the rotation speed under operating conditions. 

After the introduction this paper is organized as follows. Section 2 gives a detailed description of the structure of the proposed sensor system used to monitor the pressure profile in chalcographic printers. This section analyzes the sensor module, the heating module, the signal conditioning module, the acquisition system and the data representation. The experimental results obtained are presented in Section 3. Finally, some conclusions are drawn in the final section.

## 2. Sensor System Description

**Figure 1:**
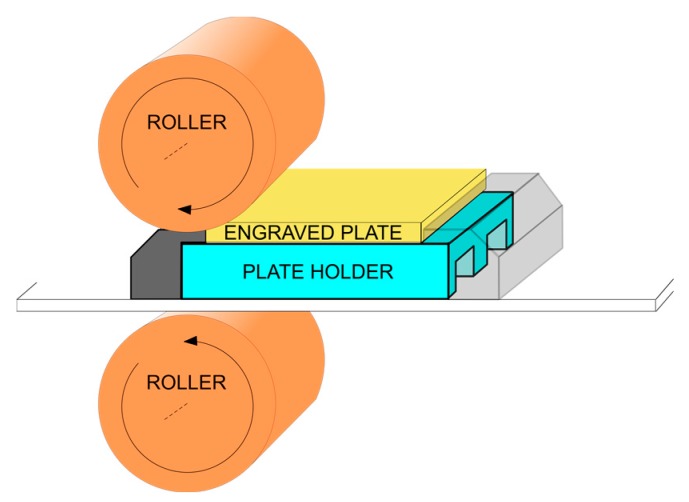
System structure of the chalcographic printer.

As a preliminary step before the proposed sensor system architecture is described, Figure 1 depicts the printer structure with a pressure profile to be measured by our system. The aim of the sensor system is to determine whether the pressure or mechanical stress distribution on the surface of the engraved plate is symmetrical and uniform, thereby ensuring the quality of the engraved printing.

In a chalcographic printer, the upper roller is a carbon steel cylinder having a diameter of 200 mm with an outer coating based on several carbon layers and a rubber blanket. The mechanical system makes the rollers rotate, which produces the movement of the structure containing the engraved plate. This plate is a flat surface of 140 × 100 mm and has a thickness ranging from 3 to 4 mm, coated with a thin layer of chrome and made of copper and sometimes nickel. 

The plate holder, located underneath the engraved plate, has been modified with the aim of placing the strain gauges. From the perspective of the sensor system, it can be claimed that the plate holder has been redesigned to sense the pressure, thereby working as a load cell. Therefore, the plate holder is the essential part of the sensor module included in the monitoring system. 

**Figure 2:**
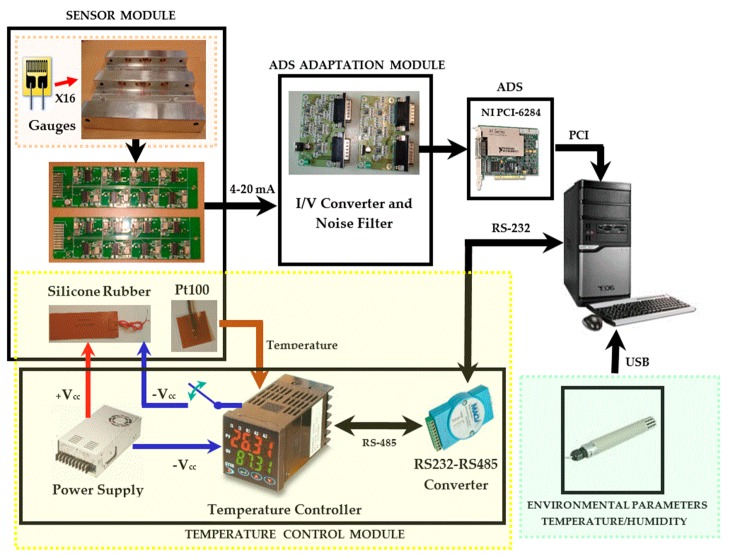
Architecture of the proposed sensor system.

Figure 2 shows the architecture of the proposed sensor system which consists of the following parts: sensor module, Acquisition Data System (ADS), ADS adaptation module, temperature control module and the sensors to capture the environmental parameters, namely temperature and humidity.

Briefly, the sensor system works as follows: the measurements obtained by the sensor module are sent to the conversion and conditioning module. This module filters and transforms the current loops into voltages before the signals are sent to the acquisition module, which is based on a commercial board installed in a personal computer (PC). The temperature control module is responsible for raising and maintaining the plate holder to the ink optimal temperature, normally 80 °C. Finally, the system includes a commercial temperature probe used to measure the environmental temperature and humidity. These parameters are of interest to the reports generated by the system. Following on, for each of the modules listed before, the specifications, design and implementation and the results obtained are detailed and analyzed.

### 2.1. Sensor Module

**Figure 3:**
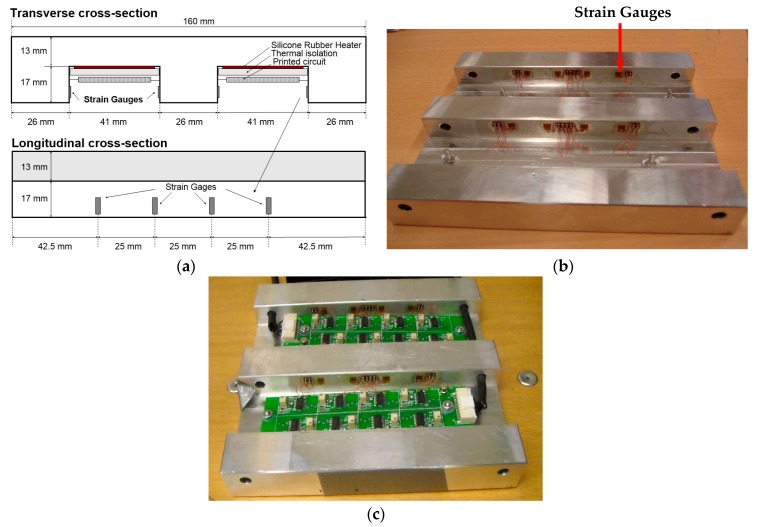
(**a**) Structure of the designed plate holder; (**b**) The plate holder (160 × 160 × 30 mm) with the strain gauges adhered to the edges of the grooves and (**c**) Signal conditioning electronics integrated on the plate holder.

Figure 3 depicts the plate holder structure designed to capture the mechanical stress distribution. As can be seen, the rectangular prism-shaped plate holder with dimensions 160 × 160 × 30 mm has two grooves and on the square edges of the grooves, 16 strain gauges have been placed (4 on each edge, see Figure 3b). Likewise, the signal conditioning modules for the strain gauges have been located on the base of the grooves (see Figure 3c).

According to the measurements taken by two load cells placed on the shaft ends of the rollers, the maximum force applied to the engraved plate is around 2000 kg. On the other hand, the linear velocity of displacement of the plate holder can vary in a range from 0.10 to 0.28 m/s. The plate holder has been made of a 7075-T7351 aluminum-zinc alloy. As a result, it exhibits a low Young’s modulus of about 72 GPa, providing strain higher than that of other metals such as steel or copper, and a high elastic limit of 400 MPa, which is higher than that of the engraved plate. This fact ensures that the holder plate can work in elastic mode.

In order to calculate the maximum mechanical stress between the roller and the plate holder ,analytical cylindrical contact force models [21,22] and the plane strain problem of a curved elastic body pressed against an elastic half-space [23,24] have been considered. Finally, applying the Hertz equations to the static contact between the roller and the plate holder yields a maximum mechanical stress of 164 MPa, for an expected force of 2000 kg (applied on the system). Clearly, this force is below the elastic limit of the plate holder. When the maximum force is exerted, the contact surface is 1.1 mm width. In practice, this width is greater on account of the engraved plate between both elements, i.e., roller and the plate holder, the machined grooves on the plate holder, the roller coating and its displacement. Therefore, the maximum mechanical stress is lower than that obtained from the Hertz equations. This is proved by the measurements taken in the final system concluding that the mechanical stress endured by the plate holder is below its elastic limit, which ensures that the system is working without permanent strains. 

**Figure 4:**
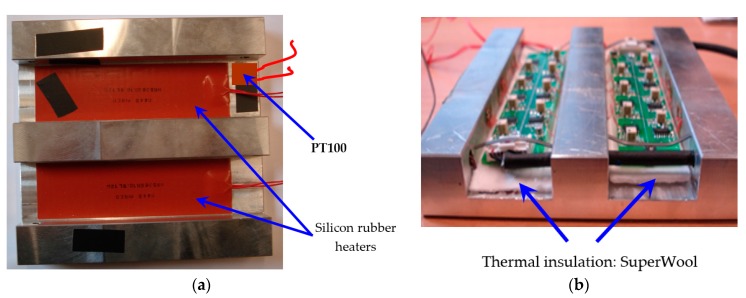
(**a**) Heating module for the plate holder: PT100 and silicon rubber heater; (**b**) Thermal insulating arrangement.

With the aim of raising and regulating the system temperature to 80 °C (see Figure 4a), silicon rubber heaters and a temperature sensor Pt100 have been installed in the grooves together with the printed circuit board that contains the signal conditioning electronics for the strain gauges. To prevent thermal drifts due to the close proximity between the rubber heaters and the conditioning electronics, and therefore, protect the electronic components from damage caused by overheating, thermal fiber (superwool) has been used for insulation of both parts. Despite its minimum thickness, this type of thermal fiber exhibits excellent insulating properties (see Figure 4b).

Considering both the Young’s modulus of the 7075-T7351 aluminum alloy (E = 72 GPa) and the previously worked out maximum mechanical stress of 164 MPa, the maximum strain produced is 2278 με [21]. The model of the strain gauge used in the design of the sensor system is the EA-13-031CE-350/E by vishay [25]. These gauges have a gauge factor of 2.1 and feature self-temperature-compensation for the 7075-T7351 aluminum alloy. Since the strain gauges are not embedded in a Wheatstone bridge configuration, this compensation is required to minimize the effects of the thermal expansion coefficient on measurement, equivalent to +17.5 με/°C. This, together with the wide range of operating temperatures, produces a maximum total strain of around +1000 με. This value is comparable with the values of the strain measured as a consequence of the mechanical stress. The thermal expansion coefficient is the same for all the strain gauges in the plate holder and hence, no error in the mechanical stress measurement is introduced. However, there is a drastic reduction in the dynamic range, which negatively influences the measurements in the signal conditioning electronics module.

A circuit based on the 4–20 mA current loop transmitter XTR105U by Texas Instruments [26] has been designed to condition the signals from each of the 16 strain gauges. The transmitter exhibits an output sensitivity of −8 μA/με. Since it is a custom design, there is no need to meet the 4–20 mA current loop standard. Bearing in mind that the transmitter XTR105U operates in the linear region for currents ranging from 2.6 mA to 24 mA, the maximum dynamic range for the measurements is 2675 με.

The system performs an autocalibration as a prerequisite before monitoring the printing process. The method of autocalibration in this work involves the measurement of the signal amplitude provided by each strain gauge when they are at rest. These amplitudes are then used as a reference to determine the variations found when the roller is rotating. Therefore, the system offset thermal drift does not introduce measurement uncertainty although it does reduce the acceptable dynamic range of the deformation.

The designed circuit has a thermal drift offset at the output of ±1.2 mA under the following condition: maximum expected temperature variations of 60 °C from the set temperature of around 20 °C to the printing maximum temperature of 80 °C. This reduces the measurement dynamic range by ±150 με from both ends of the measurement scale. Taking this into account, the system has been calibrated at a room temperature of 22 °C so that it can provide 4 mA, without exerting mechanical stress and leaving a safety margin of 1.4 mA for the offset thermal drift. This margin of 1.4 mA comes from the difference result of the difference between the 4mA setting and the linear operating limit of 2.6 mA.

The sensitivity thermal drift is extremely important since it modifies the sensitivity of each of the channels of the conditioning electronics, having an influence on the mechanical stress profile obtained. For this parameter, due to the gauge factor thermal drift (+100 ppm/°C), each channel is affected by the same amount. As a result, it will not have any effect on the measurement. The sensitivity thermal drift is also influenced by the electronic components that constitute the 4–20 mA current loop transmitters. 

With the device used in the design, a drift in the sensitivity as a function of the temperature of ±18 ppm/°C (±1σ) can be achieved. When evaluating within the operating temperature range of 60 °C and for ±3σ, the maximum sensitivity deviation is ±0.32%. Similarly, the design guaranteed maximum sensitivity tolerance due to the real characteristics of the components is ±0.3%. The combined effect of such tolerance and the thermal drift yields a maximum uncertainty of ±0.44% (±3σ) in the sensitivity, and therefore, in the mechanical stress measurements.

The analysis of the intrinsic noise generated at the output yields a value of 16 μApp, equivalent to a maximum uncertainty of ±1 με, which can be negligible. This noise was calculated over −3 dB bandwidth of 100 Hz, being the bandwidth determined by the XTR105U (Texas Instruments, TX, USA) and the electronics of the ADS adaptation module. 

### 2.2. ADS Adaptation Module

This module low-pass filters the input signal and converts the current from the 4–20 mA current loop transmitters into voltage, before being adapted to the input range of the data acquisition board.

The current-voltage conversion factor is 352 mV/mA, hence the output sensitivity will be −2.816 mV/με. Since the maximum input current is 24 mA, the maximum output voltage is set to a value of 8.45 V.

The bandwidth of the first-order low-pass filter is 160 Hz. Therefore, the final bandwidth of the system considering the response frequency of the XTR105U transmitter is on average 100 Hz, which is sufficient to capture the temporal variations of the information to measure. This fact will be proved later in the results sections. 

As in the case of the 4–20 mA current loop transmitters, the important contributions to uncertainty are produced by the sensitivity thermal drift which, for the devices employed in the design, takes a typical value of ±14 ppm/°C (±1σ). This value is slighty below that obtained with the 4–20 mA current loop transmitters of ±18 ppm/°C. As for the sensitivity tolerance due to the real characteristics of the components, a design guaranteed value of ±0.14% has been calculated. The combined effect of the ADS adaptation module and the 4–20 mA current loop transmitters introduces a maximum deviation in the sensitivity and, therefore, in the mechanical stress measurements, of ±0.53% when evaluating within the operating temperature range of 60 °C and for ±3σ. Finally, due to the high-level input signal, the electronic noise introduced by the ADS adaptation module is negligible. 

### 2.3. ADS Module

The ADS is based on the PCI-6284 data acquisition board by National Instruments [27]. This board provides 32 analog inputs with sequential sampling. The sampling rate for each of the 16 channels used to capture the information from the strain gauges is 1 KS/s. Considering a maximum plate holder displacement speed of 0.28 m/s, the spatial resolution for the measurement system is above 3 samples/mm. Both the sampling rate and the system bandwidth are sufficient to capture the variations of the mechanical stress exerted, as will be proved later. 

The acquisition board includes 18-bit ADCs. Since the input range per channel has been set to ±10 V, the quantification step is *q* = 76 μV. Taking into account the output sensitivity of the ADS adaptation module, −2.816 mV/με, results in an equivalent quantification step of −27.1 × 10^−3^ με. The Absolute Accuracy at Full Scale that was found in the datasheet is ±980 μV, which corresponds to ±0.35 με. This is even below the value introduced by the 4–20 mA current loop transmitters. Therefore, the ADS influence on the system accuracy is negligible. 

**Figure 5:**
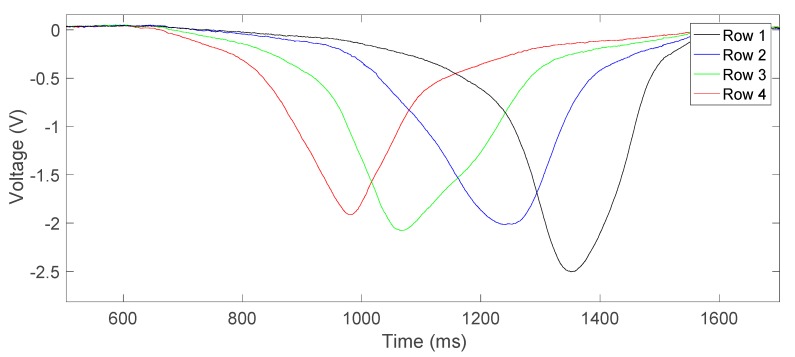
Signal captured from ADS of four consecutive strain gauges (Row 1, Row 2, Row 3 and Row 4) in the same groove. The voltage sign has been changed with respect to the voltages sampled by the ADS with the aim of reflecting the real strain.

As an example, Figure 5 shows the signals from 4 strain gauges located on the same edge of the same groove in the plate holder, captured from the ADS. The time axis is given in milliseconds while the amplitude axis is in volts. As can be seen in Figure 3a, the distance between the strain gauges is constant and takes a value of 25 mm. Therefore, the distance between maxima in Figure 5 shows that the displacement speed of the plate holder is not uniform, although this issue is not critical for the purposes of this system. The time distance between the first and the last peaks (maximum peaks) is 400 ms. This indicates that the average displacement speed in this experiment is 0.19 m/s, which is within the expected range of 0.1 to 0.28 m/s.

Considering the value for the ADS input sensitivity of −2.816 mV/με, the maximum strain values extracted from Figure 5 that occur when the contact surface of the roller and the strain gauges exactly coincide range from −700 to −900 με.

As can be observed in Figure 5, the signal voltage approximately changes following a triangular curve. In this case, the maximum error in a first-order low-pass filter takes the value of the gradient of the triangle wave. This gradient is a direct function of the the strain and the plate holder speed. The maximum error is this gradient multiplied by the filter time constant with a value of 1.59 ms = 1/(100 Hz·2π), considering a bandwidth of 100 Hz. For the signals depicted in Figure 5, a maximum error of about ±20 mV must be taken into account. This corresponds to ±7 με when measuring a strain of about 1000 με, i.e., an error of ±0.7%. This error is virtually the same for each strain gauge, assuming that they are subjected to similar strain and for a constant displacement speed of the plate holder. Therefore, the effect of this error on the measurement of the mechanical stress uniformity will be below ±0.7%, which is acceptable. 

Finally, the curves in Figure 5 apparently do not show a noise contribution. When the signals from the ADS are analyzed at rest, i.e., with the printer shut down, yielded values for the noise range from 3.6 mVpp to 5 mVpp, depending on the acquisition channel, which corresponds to strains from ±0.64 to ±0.89 με, respectively. These values are negligible, in line with the expected values after the theoretical analysis of the designed system. Therefore, the chosen bandwidth of 100 Hz for the measured system proves to be adequate for both accurately obtaining the information and effectively limiting the noise to negligible levels. 

### 2.4. Temperature Control Module

This module consists of the temperature controller aiming to maintain the plate holder temperature at a constant value. The user can select this temperature through an application installed on a PC. By using the information from the temperature sensor Pt100 and considering the temperature reference selected, the controller provides the necessary power so that the silicon rubber heater produces the heat energy to regulate the temperature to the desired value. 

**Figure 6:**
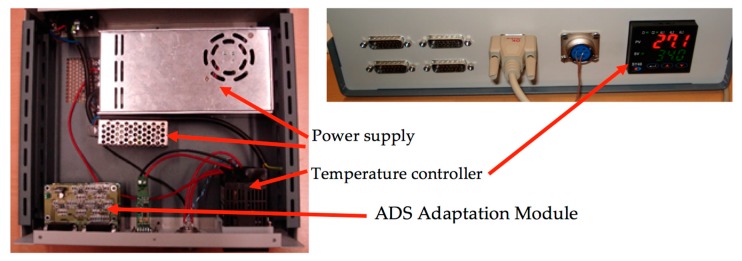
Interior and frontal part of the device integrating the temperature controller and the ADS adaptation module.

A switch mode power supply unit of 27 V and 200 W is used as a power source for the silicon rubber heaters. Finally, an RS485-to-RS232 converter allows the temperature controller and the PC application to communicate. Figure 6 shows the front and the interior of the device, which incorporates the temperature control system and the ADS adaptation modules.

The resistance heaters embedded on the plate holder exhibit a nominal resistance of 10.9 Ω. When they are powered with a voltage of 27 V from the power supply unit, the dissipated power for each resistance heater is 67 W. This power is sufficient to raise the temperature to 80 °C in approximately 15 min.

## 3. Results

**Figure 7:**
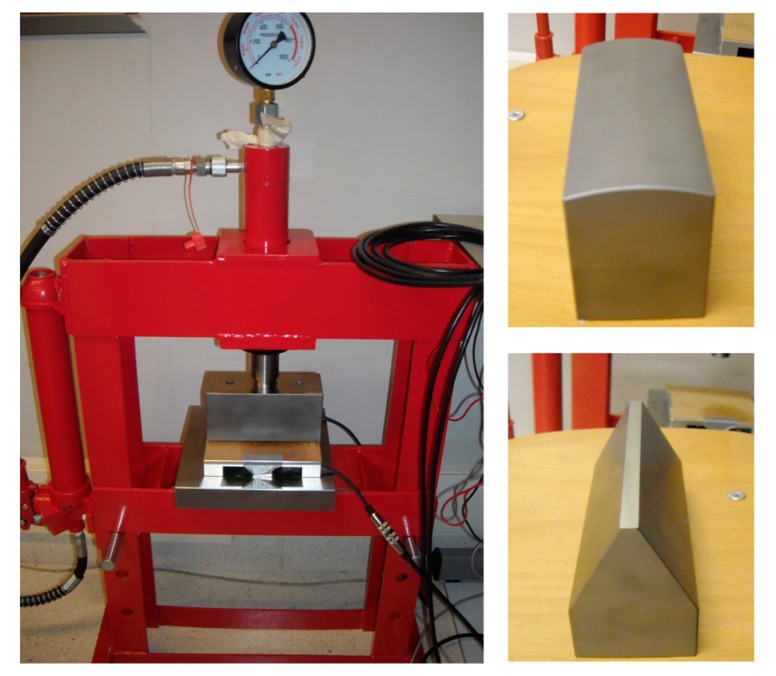
Hydraulic press used in the tests.

With the aim of verifying the operation of the system, several tests have been conducted. Before performing the tests on the chalcographic printer, a hydraulic press, designed to provide a pressure of 6000 psi, was used as a test bench. Likewise, several tools were developed to exert pressure over the sensor on different points. In this way, the strain gauge deformations were qualitatively measured by using a graph paper template to discover the coordinates of the points on the plate holder where the pressure is exerted. Figure 7 shows the hydraulic press with the sensor system integrated and the tools developed.

The tests conducted by using the hydraulic press demonstrated that it is not possible to calibrate the response of the different strain gauges. For that aim, it is necessary to ensure high accuracy of the position of the tools over the plate holder, which is not feasible with the available equipment. Failing that, the uniform response of the strain gauges has been guaranteed during the design phase by both calculating the uncertainty introduced by the electronics and taking into account the characteristics of the strain gauges. The total uncertainty introduced by both contributions, calculated in Section 2, is ±0.53% (±3σ). In addition, since the process of placing the plate holder is not perfect, the contribution to the uncertainty due to the misalignment of the strain gauges was also considered. The strain gauges placing was carried out by the technical support of the firm called Vishay, who ensured a maximum angular deviation of ±5° with respect to the perpendicular to the plane of the plate holder. Considering this angular deviation, the maximum relative error for the deformation suffered by the strain gauges is +1% [28], with this value being the maximum difference in sensitivity among the different strain gauges. Therefore, under the design conditions stated above, the sensitivity error of the system ranges from −0.53% to +1.53%, which is significantly below the adjustment threshold of ±10% (see Section 3) so that the roller can be considered to be aligned.

### 3.1. Tests on the Chalcographic Printer

As has already been mentioned, the press consists of two rollers which move the plate holder synchronistically. The upper roller has a screw at both ends, which allows both control and adjustment of the pressure used to make the impression. 

In this type of press, the adjustment is made by using a trial-and-error approach to verify the printing quality. Therefore, several impressions are made resulting in a waste of material. The proposed system allows the adjustment to be made without wasting either paper or ink. This is simply done by moving the roller over the plate holder. 

Several tests were carried out with an aligned roller and with the roller intentionally misaligned to different degrees: the roller with misalignment from the left and from the right, and small or significant misalignment. For each test and for each strain gauge, the deformations were measured and registered, and, finally, the printing quality was evaluated by staff with printing skills and experience.

**Figure 8:**
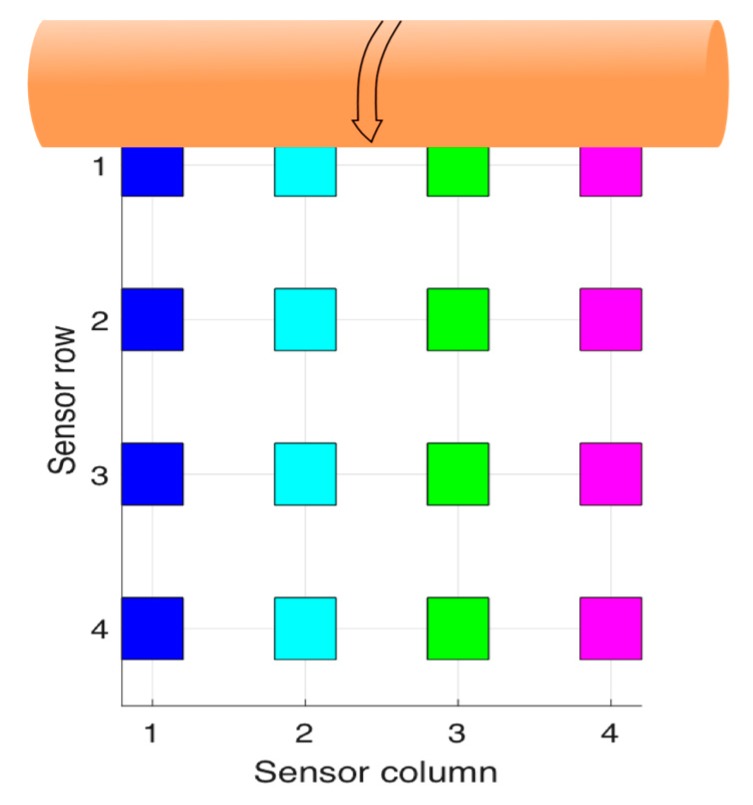
Strain gauges position with respect to the roller movement direction.

**Figure 9:**
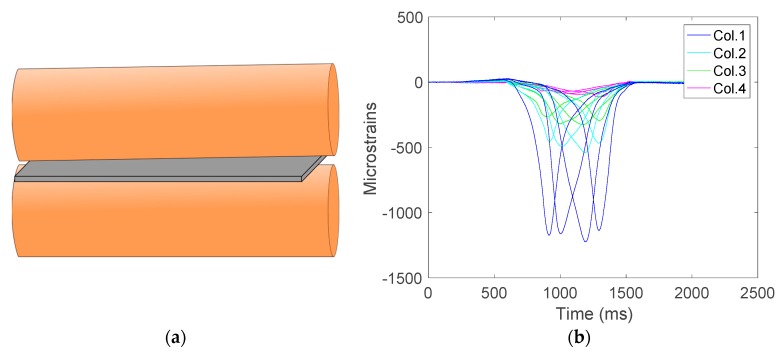
(**a**) Diagram when the roller is in misalignment and (**b**) corresponding signal captured.

**Figure 10:**
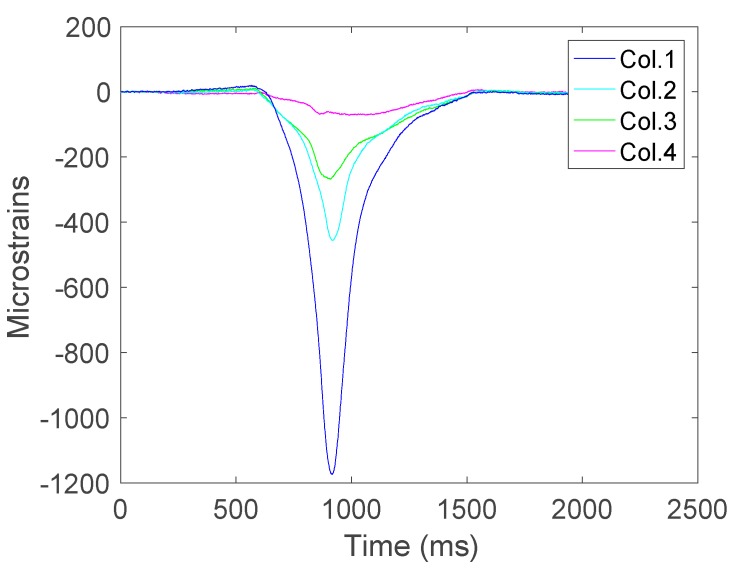
Deformations of the strain gauge on row 3.

**Figure 11:**
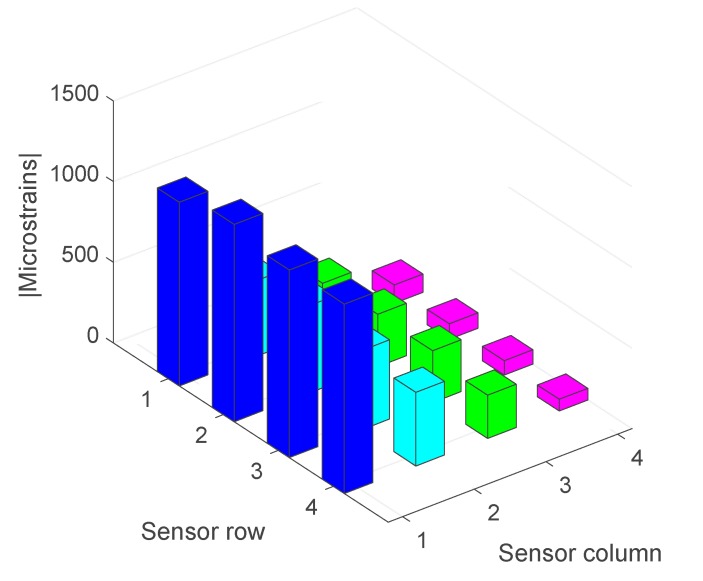
Maximum deformations of the strain gauges when there is a significant misalignment (absolute values).

**Figure 12:**
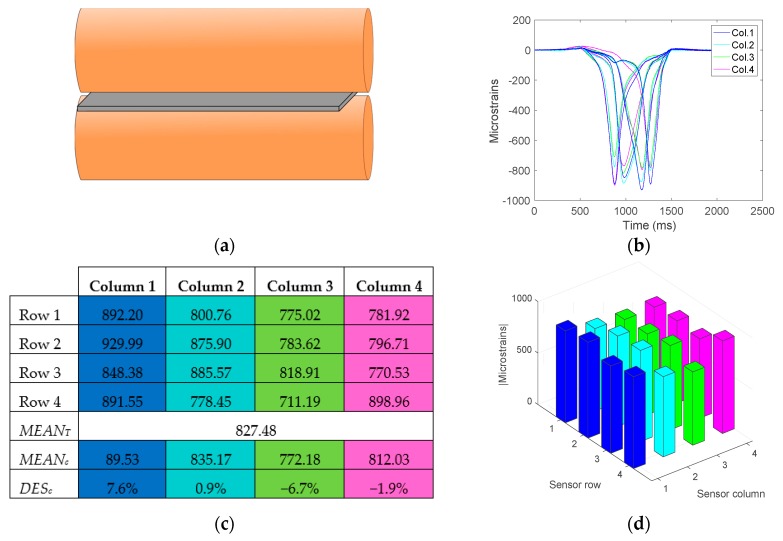
Test with aligned roller. (**a**) Diagram of the roller position, (**b**) Strain gauge deformation, (**c**) Maximum absolute values of the gauge deformations, means and deviations. (**d**) Representation of the maximum absolute values.

**Figure 13:**
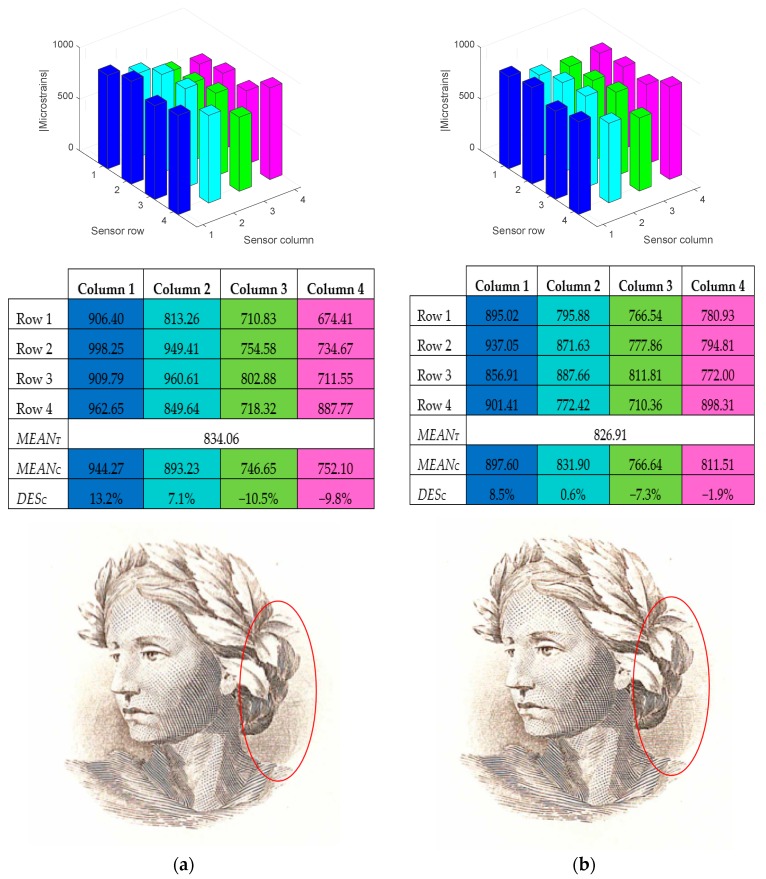
Engraving comparison. (**a**) Roller with minor misalignment and (**b**) aligned roller.

In order to identify the acquired data, Figure 8 depicts the spatial arrangement of the gauges with respect to the movement direction of the roller. Row 1 is, therefore, the first one to be subjected to the pressure exerted by the roller. The measured deformations for each strain gauge are shown in Table 1 and Table 2 and Figure 9, Figure 10, Figure 11, Figure 12 and Figure 13, in which the same colour code has been used.

#### 3.1.1. Test with Misaligned Roller

Figure 9a shows the roller position for this test, and Figure 9b shows the deformations caused by the roller and the time when they happen. In Figure 9b, the *x*-axis represents the time in milliseconds (ms) and the *y*-axis the deformations (με).

As can be seen in Figure 9, the deformations suffered by each strain gauge on the same column have the same order of magnitude and the maxima happen sequentially in time, synchronized with the displacement of the roller. 

To better illustrate the roller misalignment, Figure 10 depicts the deformation measurements corresponding to the four strain gauges in row 3. In this figure, it is noticeable that the peak values happen simultaneously but the higher values are obtained from the area subjected to more pressure. 

In order to determine the misalignment, it has been found that the best indicators are the peaks reached by each strain gauge. Hence, this has been the data used in the analysis. For the sake of clarity in the graphical representations, the maximum values depicted in the figures are given in absolute values although the deformations have a negative value. 

Figure 11 shows the absolute magnitude of the maximum deformations suffered by each strain gauge on the sensor $\left|S_{RCm\text{á}x}\right|$. It is observed that the deformation is at a maximum in column 1 and minimum in row 4, which clearly indicates a roller misalignment. 

Table 1 shows the maximum absolute values for the deformations of each strain gauge in με and the mean value of them. The mean value of the deformations is also included, as it will be used in the final section.

The total mean value, *MEAN_T_*, is worked out by averaging the maximum values of the deformations captured by the strain gauges as follows:

(1) $$MEAN_{T}=\frac{\sum_{R}\sum_{C}S_{RCmax}}{16}$$

The condition of alignment is determined from the relative differences with respect to the mean value. For such purpose, the mean value of each column, *MEAN_Ci_*, is calculated by using Equation (2) and then the deviation from the total mean value, *DES_Ci_*, is determined by using the expression (3).

(2) $$MEAN_{Ci}=\frac{\sum_{R}S_{Rimax}}{4}$$

(3) $$DES_{Ci}=\frac{MEAN_{ci}-MEAN_{T}}{MEAN_{T}}\cdot 100$$

Table 2, shows the deviations corresponding to the previous test considering a significant misalignment.

#### 3.1.2. Test with Aligned Roller

The following test (Figure 12), illustrates an example of impression when the roller is in alignment. 

It can be concluded that, after performing multiple tests, the roller can be considered to be aligned when all the deviation values *DES_Ci_* are below ±10%. This value has been determined by consulting with staff with expertise in printing about the quality of the images produced for the different degrees of deviation. 

Figure 13a,b shows two examples where an engraving has been made with a roller with slight misalignment and an aligned roller, respectively. The differences are hardly noticeable for a non-expert in the domain of printing techniques, since the roller misalignment is close to the adjustment threshold indicated above. However, the printing expert considers valid the impression on the right (Figure 13b), which allows choosing a value for the adjustment threshold of ±10% for the *DES_Ci_* parameter, as stated previously.

## 4. Conclusions

This paper presents a novel sensor system aimed at on-line monitoring of contact pressure with application to the field of chalcographic engraving testing. The designed system looks at the contact pressure distribution between a cylinder and a flat surface. In order to monitor the contact pressure profile over the engraved plate, the proposed system uses an electronically integrated strain gauge-based load cell, which consists of a grid of 16 strain gauges arranged in a 4 × 4 matrix.

The monitoring system developed can be applied to any printing equipment, e.g., flat-flat and cylinder-flat, provided that the contact pressure profile over the engraved surface is required.

The results obtained within the experimental setting prove that the developed prototype operates effectively and efficiently. The representation of the contact pressure profile can be obtained while the printer is on production, i.e., on-line. This profile allows information about the cylinder-plate alignment to be obtained for each impression.

To conclude, the proposed system constitutes a remarkable improvement in the printing process in terms of cost savings, in materials and avoiding breakdowns, as well as in the quality and reproducibility of the engravings under similar printing conditions over time.

## Figures and Tables

**Table 1:** Maximum deformations suffered by each strain gauge, given in με, under significant misalignment on the left and the mean value of the maxima.

	Column 1	Column 2	Column 3	Column 4
Row 1	1138.55	468.78	294.93	113.73
Row 2	1223.59	541.81	325.73	95.34
Row 3	1163.09	491.48	321.36	88.76
Row 4	1174.24	456.43	268.16	72.23
*MEAN_T_*	514.89			

**Table 2:** Maximum deformations (με) suffered by each strain gauge in test 1: significant misalignment on the left and total mean value of the maxima.

	Column 1	Column 2	Column 3	Column 4
*MEAN_C_*	1174.87	489.63	302.54	92.52
*DES_C_*	128.2%	−4.9%	−41.2%	−82.0%
